# Engineering Micromechanical Systems for the Next Generation Wireless Capsule Endoscopy

**DOI:** 10.1155/2015/741867

**Published:** 2015-07-15

**Authors:** Stephen Woods, Timothy Constandinou

**Affiliations:** Centre for Bio-Inspired Technology, Department of Electrical and Electronic Engineering and Institute of Biomedical Engineering, Imperial College of Science, Technology and Medicine, London SW7 2AZ, UK

## Abstract

Wireless capsule endoscopy (WCE) enables the detection and diagnosis of inflammatory bowel diseases such as Crohn's disease and ulcerative colitis. However treatment of these pathologies can only be achieved through conventional means. This paper describes the next generation WCE with increased functionality to enable targeted drug delivery in the small intestinal tract. A prototype microrobot fabricated in Nylon 6 is presented which is capable of resisting peristaltic pressure through the deployment of an integrated holding mechanism and delivering targeted therapy. The holding action is achieved by extending an “anchor” spanning a 60.4 mm circumference, for an 11.0 mm diameter WCE. This function is achieved by a mechanism that occupies only 347.0 mm^3^ volume, including mechanics and actuator. A micropositioning mechanism is described which utilises a single micromotor to radially position and then deploy a needle 1.5 mm outside the microrobot's body to deliver a 1 mL dose of medication to a targeted site. An analysis of the mechanics required to drive the holding mechanism is presented and an overview of microactuators and the state of the art in WCE is discussed. It is envisaged that this novel functionality will empower the next generation of WCE to help diagnose and treat pathologies of the GI tract.

## 1. Introduction

Inflammatory bowel disease (IBD) is a long-term condition of the GI tract. The most common forms of this inflammatory condition are Crohn's disease and ulcerative colitis which can affect the colon and small intestine. These autoimmune diseases can be treated with antibiotics; however diagnoses can be difficult and treatment can lead to side effects such as dysbacteriosis. An approach to diagnose conditions of IBD is wireless capsule endoscopy (WCE). WCE has become a valuable tool for the diagnosis of pathologies of the gastrointestinal (GI) tract [1]. These small pill-sized cameras allow the gastroenterologist the ability to diagnose pathologies in the small intestinal tract, which is the most difficult section of the alimentary canal to reach. The pill-sized cameras take pictures of the intestinal wall and relay them back to a recorder for evaluation at a later date.

An early example of a swallowable WCE is the M2A developed by Given Imaging Ltd. in 2000 [2]; it was renamed PillCam SB(R) in September 2004. The PillCam was specifically designed to overcome the problem of examining the small intestine. The capsule which is 11.0 mm in diameter and 25.0 mm long comprises a complementary metal oxide semiconductor (CMOS) camera, four illuminating light emitting diodes (LEDs), a radio frequency (RF) module, and a power supply. WCE has now become the gold standard for examining the GI tract and there are a number of WCEs available commercially for this purpose; a detailed review of commercial WCE systems can be found in [3, 4].

WCE systems are generally restricted to diagnostic use as the availability of onboard space for surgical tools or medication limits the ability to treat pathologies such as ulcerative colitis [5]. This inability to deliver therapy to an area of interest in the GI tract leaves only the curative options of administering large quantities of drugs or surgical intervention.

The paper is organised as follows.  Section 2 describes the state of the art in WCE systems, Section 3 presents the background to actuators employed to drive surgical tools, Section 4 presents a novel method for targeted drug delivery and a method to overcome natural peristalsis in the GI tract, Section 5 presents the design, analysis, and prototyping of a novel holding mechanism, Section 6 discusses the performance of a functioning prototype holding mechanism, and Section 7 concludes this work.

## 2. State of the Art

Conventional WCE systems are passive medical devices which have been focused on imaging the GI tract; however for the next generation to be useful surgical tools they will require active mechanical parts to enable them to carry out tasks such as performing a biopsy or electrosurgery while resisting natural peristalsis.

### 2.1. Resisting Peristalsis

In order to overcome the limitations of conventional WCE researchers have been exploring methods of increasing a capsule's functionality such as methods of resisting natural peristalsis [6–8] or navigation through the GI tract [9–12]. The paddling based microrobot developed by Park et al. (2007) [13] was developed specifically for navigating the GI tract. This method of locomotion uses a leadscrew engaged with six radially positioned legs to propel a microrobot forwards. A method for anchoring to the intestinal wall which reduces the risk of injury to the intestinal tissue is proposed by Glass et al. (2008) [14]. They propose a three-legged anchoring mechanism which utilises micropatterned adhesives for resisting peristalsis. The anchoring mechanism consists of three axially aligned legs equally spaced around the capsule. Each leg is connected to a cylindrical pulley which is free to rotate. One end of a cable is secured to the capsule while the other end is secured to the upper end of the leg. The normal state of the cables is relaxed with the legs in the closed position. The leg pulleys are connected to the base of the capsule via rubber springs. When the anchoring mechanism is activated micromotors or shape memory alloy (SMA) wires pull the cables; this results in a torque being applied to the pulleys which rotate the legs opening them outwards. At the same time the rotation applies a torque to the rubber spring resulting in an opposing torque; this counter torque is the means of returning the legs to the closed position when the cable torque has been removed.

An alternative clamping system for clamping to the wall of the GI tract for the purpose of long-term pH monitoring has been proposed by Menciassi et al. (2005) [15]. The clamping system utilises three grasping units which extend forwards from the capsule and can be activated to clamp to the intestinal wall. The clamping mechanism is a combination of two subsystems, one system to operate the protrusion and return of the grasping unit and another system to operate the opening of the grasping unit. The clamper unit slides in a groove which runs parallel to the capsule's body and is the vehicle which moves the grasping unit. The clamper unit is driven by thermally activating a 100 *μ*m diameter SMA wire which is housed in a trench that runs alongside the groove. The wire has been coiled to generate the large forces required to slide the clamper unit forwards to bring it into contact with the intestinal wall. The grasping unit comprises two arms made from SMA. The arms are normally in a closed position which is achieved by virtue of a flexure joint which biases the arms. The arms are activated by heating a 20.0 mm long by 50 *μ*m diameter SMA wire which has been fixed into holes in the arms. The heating causes the wire to contract which opens the arms.

Current WCE devices lack the ability to control their speed and direction relying on natural peristalsis to move them through the GI tract. A solution that overcomes this problem is the robotic legged locomotion device developed by Valdastri et al. (2009) [16]. The 12-legged endoscopic capsular microrobot features two sets of six legs integrated into the capsule. These propel the device through the GI tract and help to uniformly distend collapsed colon tissue making visualisation easier. The legs are operated via their connection to a nut which moves axially up and down a leadscrew. The set of legs simultaneously open and close as the nut translates the leadscrew, with each set of legs being independently controlled by a motor and gearbox. The number of legs distributes the contact force over the colon wall reducing slippage.

The procedure of inspecting the lining of the oesophagus using a flexible endoscope is routine; however it is also a very uncomfortable procedure for the patient. Tognarelli et al. (2009) [17] propose SMA flat springs to halt the progress of an oesophageal WCE for the purpose of inspecting the oesophagus lining. The transit time through the oesophagus is rapid; therefore a stopping mechanism must have a quick response time. The 11.0 mm in diameter by 31.0 mm long capsule developed by Tognarelli et al. [17] can deploy a stopping mechanism suddenly to halt its progress through the oesophagus. The stopping mechanism consists of three equally spaced SMA flaps, a DC brushless motor, a pulley, a gear set, and three 0.15 mm diameter Kevlar wires. Rather than being triggered by the Joules effect the SMA legs have been set in an open position to take advantage of the superelastic properties of the material. The DC motor is connected to the gear set and pulley and is used to wind the legs into a cavity in the capsule body by virtue of the Kevlar wire which connects the tip of the legs to the pulley. When the wire is released the flaps return to their original open position and halt the progress of the capsule.

### 2.2. Drug Delivery

Increasing a capsule's functionality to include the means of delivering medication to a site of interest offers great advantages to patients. A commercial system developed by Phaeton Research attempts to deliver drugs to a specific location in the alimentary canal. The Enterion capsule [18] is administered with a standard volume of water (240 mL) [19] and relies on natural peristalsis to move it through the body. The device is manufactured from FDA approved plastics and has a capacity of storing 1 mL of medication in a drug reservoir in either a liquid, powder, or solid form. The medication is loaded by simply removing a bung at the rear of the capsule and filling up the drug reservoir. The medication is administered through the use of a compressed spring which when triggered operates a cylindrical piston. The piston expels the medication through the rear aperture at the same time as ejecting the bung. The spring is held in position by an anchoring mechanism which consists of a thin thread connected to the piston and to a heating element which is mounted on a circular printed circuit board. The anchoring mechanism works by heating the thin thread until it becomes weak and breaks releasing the spring and hence operating the piston. The heating element which is a resistor is triggered by an externally applied radiation. When the device passes through an electromagnetic field at the frequency an onboard receiver is tuned to, the induced current powers the resistor which heats the thin thread. The 1 mL of medication can be expelled at a target region in the GI tract such as the jejunum, ileum, ascending colon, or descending colon. It cannot target specific pathogens such as tumours or ulcers because it releases its payload as a bolus form. This has the effect of spreading the medication over a section of lumen as the capsule is under constant movement from peristalsis and has no means of stopping and holding its position.

The InteliSite is a drug delivery system manufactured by Innovative Devices LLC [20]. It opens ports on the side of the device releasing the drugs into the GI tract. It has a 1 mL reservoir capable of being loaded with either a liquid or a powder drug formulation. The drugs are administered when the capsule is exposed to a radio frequency magnetic field. The magnetic field causes two SMA wires to heat up and straighten out. This action causes an inner sleeve which has a series of slots in it to rotate aligning it with a series of slots in the outer surface. This action allows the drugs to be released into the GI tract. The device has no means to propel the drug from the capsule into the GI tract; instead it relies on the natural turbulence generated from the capsule passing through the GI tract to disperse the medication.

Philips Research has developed IntelliCap [21], an intelligent pill for electronically controlled drug delivery in the GI tract. This device uses a micropump to propel the medication into the GI tract. The IntelliCap incorporates a microprocessor, battery, pH sensor, temperature sensor, RF wireless transceiver, fluid pump, and drug reservoir. A microprocessor controls the delivery of the drug through the internal fluid pump which can disperse the medication in different delivery profiles such as burst, progressive release, or a multilocation dosing.

The telemetric capsule developed by Lambert et al. (1991) [22] has an interchangeable tip used for either aspirating or releasing a liquid into the GI tract. The tip is controlled by a magnetic switch which is activated by a permanent magnet being brought into close proximity to the capsule (closer than 15 cm). The switch triggers a microfurnace to heat up a strip of plastic which breaks after two seconds causing a clip to open; this releases a compressed spring which starts the delivery. The plastic strip is destroyed each time the capsule is deployed and needs replacing. The drug delivery tip utilises an inflatable elastic reservoir to store 1 mL of medication. A port on the side of the capsule allows for easy filling of the reservoir using a syringe; a tight Silastic joint prevents the medication from escaping. When the mechanism is activated the piston moves forward opening the way for the medication to flow through, propelled from the contraction of the expanded reservoir, and delivering the medication into the GI tract.

Karargyris and Koulaouzidis (2013) [23] propose a diagnostic capsule which provides positional information while navigating the GI tract. The system, which is based on previous work carried out by Bourbakis et al. (2010) [24], utilises two positional sensor wheels as opposed to the one wheel approach proposed by Lambert et al. (1991) [22]. The capsule's two sensor wheels are mounted on soft spiral springs. The springs extend the wheels from the capsule's body so that they make contact with the GI tract wall. Frictional forces between the intestinal wall and the wheels cause the wheels to rotate. The rotating wheels emit electromagnetic peaks which are received by precision sensors on the control board of the capsule allowing the progress of the capsule to be monitored.

### 2.3. Microbiopsy Actuators

It is challenging to diagnose pathologies of the small intestinal mucosa; however performing a biopsy can diagnose pathologies early such as in the diagnosis of small intestinal Crohn's disease [25]. To this end, researchers have been extending the capabilities of WCE to include actuators for the purpose of obtaining a tissue sample from the intestinal tract.

Park et al. (2008) [26] have developed an actuator for small intestinal biopsy which can be integrated into a conventional WCE. The microactuator has been manufactured through a LiGA process. The dimensions are 10.0 mm in diameter by 1.8 mm thickness. The actuator comprises three main component parts: a microspike for taking the biopsy, a torsion spring actuator, and an SMA heating wire triggering mechanism. The microspike is propelled forwards and backwards through the operation of a slider-crank mechanism. The slider crank comprises a connecting rod which is attached to a torsion spring at one end and the microspike at the other. A polymer string holds the torsion spring in position by anchoring it to a series of small fixing posts. This fixing method allows the polymer string to be routed across the PCB mounting board and across an SMA heating wire. When a current is applied to the SMA wire it heats up and melts the polymer string and hence releases the torsion spring which in turn propels the microspike forwards and then backwards to the stored position.

Kong et al. (2005) [27] have developed a rotational microbiopsy module which has been integrated into a WCE specifically to perform a biopsy of the small intestines. It is a compact design with a diameter of 10.0 mm and a thickness of 2.0 mm. The thickness is approximately 10% thicker than the design proposed by Park et al. (2008) [26]. The module utilises an eccentrically mounted razorblade configuration to remove a section of the GI wall. The release of a torsion spring causes the tissue-cutting razorblade to protrude from the side wall of the capsule. The blade rotates 120 degrees at a force of 10 N removing a sample of the wall as it rotates. The continued rotation of the blade causes the sample to be sealed into the WCE. The razorblade trigger mechanism is a paraffin block which restrains the blade against the torque from the torsion spring. The blade is released when the WCE receives a trigger signal causing the paraffin block to melt at 42°C. The low melting temperature ensures the intestines remain undamaged. The power requirement to operate the trigger was measured at 300 mA for 1.7 seconds and it is intended that it will be supplied by an onboard battery.

A self-propelling endoscopic system with a miniature manipulator mounted at the front is proposed by Peirs et al. (2000) [28] for retrieving a biopsy sample from the intestinal wall. The device uses two clamping modules which utilise a series of perforations to hold the intestinal wall through an applied vacuum. The modules are connected by an expansion/contraction bellow which allows the clamping modules to move independently. A hydraulically controlled Stewart platform with 3 degrees of freedom (DOF) has been utilised to position the micro tools. The hydraulic power required to drive the actuators will be supplied by an umbilical cord.

### 2.4. SMA Based Actuators

SMAs are increasingly being used by researchers as actuators to increase the functionality of WCEs as they can take advantage of their superelastic properties which allow large amounts of deformation to take place without permanently damaging the material.

Gorini et al. (2006) [29] propose the use of SMA wires to operate an actuator for a six-legged endoscopic capsule. The proposed miniaturised leg mechanism is to be integrated into a WCE for the purpose of locomotion in the GI tract. The mechanism comprises three main components: a PEEK prismatic support with dimensions of 3.4 mm × 25.0 mm × 3.2 mm, the leg, and the SMA wire system. The operation of the leg is through the activation of two 100 *μ*m diameter SMA wires connected to a brass micropulley. The right-hand wire is wrapped clockwise around the pulley and the left-hand wire is wrapped anticlockwise around the pulley, with the ends of the wires fixed to the prismatic support. Applying a current of 360 mA and 10 V for a period of 4 s to the left-hand wire causes the wire to contract by 6% and rotate the pulley approximately 135 degrees. After a period of cooling the right-hand wire is activated bringing the leg back inside the prismatic support. The degree of leg rotation is dictated by the length of contraction of the 100.0 mm long SMA wire; however the geometry of the prismatic support limits the length of wire which can be used. This issue has been overcome by the addition of glass shafts mounted in the prismatic support which allow the wire to be wrapped around them without shortcutting the circuit. The SMA wire is 40.0 mm longer than required to fully open the leg as compensation for the resistance from the antagonist SMA wire during the rotation of the pulley is required.

A two-way linear actuator mechanism driven by SMA springs is proposed by Kim et al. (2005) [30] for the purpose of locomotion in capsule-type endoscopes. The 33.0 mm long by 13.0 mm diameter capsule is intended to navigate the GI tract by means of four sliding clamps which have been arranged around the outside surface of the capsule body. The four sliding clamps carry microhooks which protrude from the capsule by 400–600 *μ*m. The purpose of the clamps and hooks is to replicate the motion of insects such as the earthworm by mimicking its setae. The 180 *μ*m diameter angled hooks are similar to those reported previously by Lee et al. (2004) [31]. The capsule moves forwards through a sequence of independent clamp movements which are driven by four two-way linear actuators. The two-way linear actuators comprise two SMA springs which are connected to either side of the clamp and are activated independently through the Joules effect. When the forward spring is contracted for a period of 2 s it pulls the clamp forwards at which point the clamp grips the surface of the GI tract. The forward spring is allowed to cool for a period of 5 s before the rear spring is contracted. The contraction of the rear spring pulls the capsule forwards with a maximum stroke length of 7.5 mm. This sequence is repeated with each sliding clamp being activated in turn to propel the capsule through the GI tract at a rate of 110 mm/min. The SMA springs reach a maximum operating temperature of 70°C with an applied current of 400 mA and a voltage of less than 2 V.

Table 1 is a comparison of the capsules discussed and their actuator method. The positions marked with a “—” signify that the feature is unavailable or that details relating to the module's function have not been published in the literature. As can be seen from Table 1 the WCEs have many different modules; however all possible modules are not contained in any one capsule.

## 3. Background

To perform a surgical task a degree of mechanical movement is required of the tools. For example, a needle must move forwards to pierce tissue or the jaw of a clamp must close to obtain a sample [32]. The kinematics of the tools performing the curative treatment can be achieved through the application of actuators. There are a number of actuators available such as SMA actuators, piezoelectric actuators, and micromotors. Choosing the correct actuator for a given task such as holding a WCE against natural peristalsis requires the consideration of a number of factors such as the available space, the type of movement, and the peak power consumption.

### 3.1. Piezoelectric Squiggle Motor

An actuator solution for controlling a holding mechanism could be the use of a piezoelectric linear motor to drive the mechanism directly. These motors use the piezoelectric effect to drive the actuation. The piezoelectric effect is the change in a material's geometry due to an applied electric charge or a mechanical stress changing a solid's shape which will result in a proportional electrical charge [33]. There are a number of issues with piezoelectric actuators such that they require displacement amplification mechanisms to obtain a useful stroke length and that they also require large voltages (100 V) to activate them [28].

**Figure 1:**
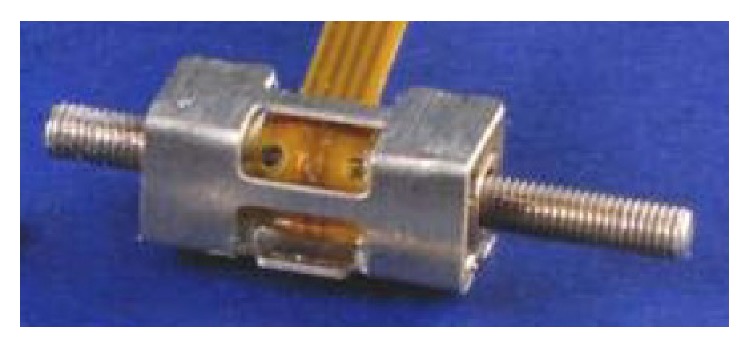
Piezoelectric Squiggle motor.

Nonconductive materials such as quartz (SiO_2_) exhibit the piezoelectric effect and can be utilised in such applications as displacement transducers. The Squiggle piezoelectric linear motor manufactured by New Scale Technologies [34] utilises the piezoelectric effect to vibrate a leadscrew causing it to travel forwards and backwards (Figure 1).

The Squiggle motor (SQL-RV-1.8-6-12) is one of the smallest piezoelectric motors in the world with a package size 6.0 mm long by 2.8 mm square and a leadscrew 12.0 mm long. The motor has a high positioning resolution of 0.5 *μ*m and a stall force (4.5 V input) of 0.5 N which makes it a good candidate for operating a holding mechanism.

An alternative to the piezoelectric Squiggle motor is a micromotor. Micromotors could potentially be used to drive the holding mechanisms directly. An examination of the latest available technology in micromotors shows that there are a limited number of micromotors on the market which could fit within the package size of the capsule and have a useful torque capability at a low RPM.

### 3.2. Micromotors

**Figure 2:**
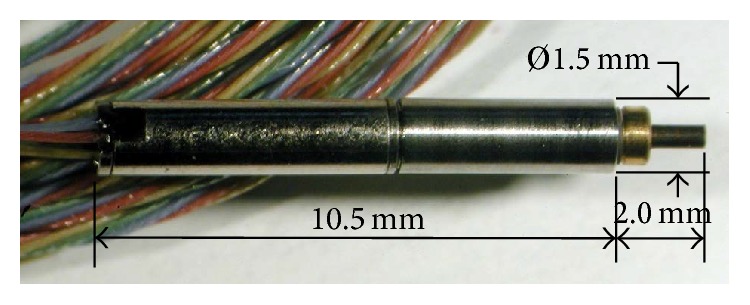
Namiki four-stage geared micromotor.

A micromotor which appears to have the required specifications is the *Ø*1.5 mm × 12.5 mm long geared micromotor manufactured by Namiki (number 10-010) [35] (Figure 2).

The four-stage geared micromotor operates at 60 mW and runs at 76 RPM; this produces a stall torque of 1.6 mNm. The Namiki motor can be compared with the specifications of three alternative motors: a *Ø*6.0 mm × 3.75 mm long motor manufactured by Maxon Motor (EC 6) [36], a *Ø*1.9 mm × 10.82 mm long micromotor manufactured by Faulhaber (02/1) [37], and the Squiggle motor. An important criterion is the micromotor's maximum power output. A comparison of the four motors using a merit index to compare the power output against the motor's volume (Table 2) shows that the Squiggle motor is far superior to the other motors as the higher the merit index is the more powerful the motor is for its size. However the Squiggle motor operates in a linear orientation compared to the rotational output of the micromotors.

The Namiki micromotor can be evaluated for its ability to perform the task of moving a 0.5 g mass of stainless steel. The stainless steel would replicate the mass of the holding mechanism and represent 10% of the total weight of the microrobot.

The torque required to move the mass of stainless steel can be calculated using the following equation:

(1) $$\begin{array}{c} T=I_{o}\alpha , \end{array}$$

where *α* is the angular acceleration and can be calculated at 265.3 rads/s^2^ from the motor's performance data and *I*_*o*_ is the moment of inertia which can be expressed as *mk* ^2^, where *m* is the mass concentrated at a radius, the Radius of Gyration (*k*), which would be 5.0 mm. This concentrated mass would be a worst case scenario as it assumes the total volume to be moved is positioned at the extreme limit of the microrobot.

The results of the initial torque evaluation show that a torque of 3.32 *μ*Nm would be required to operate the holding mechanism. Clearly the 1.6 mNm capacity of the Namiki micromotor would be sufficient to drive the mechanism; however consideration of losses due to the inherent system's friction would also be required.

### 3.3. SMA Actuators

SMAs can be used as an alternative to micromotors. The most commonly used SMA is superelastic nitinol which is an alloy of approximately 50% nickel and 50% titanium. Nitinol has superelastic properties which allow large amounts of deformation to take place without permanently damaging the lattice structure of the material. This allowable strain makes it possible to create super springs which can be triggered by the Joules effect. The disadvantages of using SMAs are that they require high power consumption and that they can affect their environment as a result of heating. Also they are unable to fully recover their original configuration after cooling due to twinning of the atomic crystal [38]. This can be overcome by detwinning the lattice through the use of a secondary process which would apply a bias to the spring.

SMA wire could be employed to drive a holding mechanism. The wire could be orientated in a pulley configuration minimising volume and attaining a mechanical advantage from the pulley. The approximate relationship between the pulley's radius (*r*) and the length (*L*) of wire required to rotate the pulley through an angle (*θ*) has been determined by Gorini et al. (2006) [29] and is given by

(2) $$\begin{array}{c} L=\frac{\pi r\theta }{0.04\cdot 180}. \end{array}$$

The 0.04 represents a 4% contraction of the wire due to the Joules effect. Applying the formula to a pulley with a radius (*r*) of 0.9 mm and a required rotation (*θ*) of 90 degrees results in a theoretical length (*L*) of the wire being 35.3 mm. The actuator mechanism would require a longer wire to operate efficiently as the theoretical model does not take into account the friction in the system and the drop in performance of the wire due to multiple operations. Also the wire would need to overcome the resistance imposed by a second wire which would be required to return the pulley to its original configuration. A consideration of the difficulty in manufacturing and assembling such small and complex components would need to be taken into account when designing such a mechanism.

### 3.4. Actuator Characteristics Comparison

The various actuator mechanisms described offer a diverse range of potential solutions to control a holding mechanism, with each actuator system having a number of advantages and disadvantages. To present a clear overview and to help understand the various options an evaluation matrix has been created using a set of criteria relating to the actuators' characteristics (Table 3). The matrix gives a quantitative assessment of the performance of each method.

The evaluation matrix highlights the problems associated with completing a task such as controlling the opening position of a holding mechanism within the GI tract. The linear movement of piezoelectric actuators and SMA actuators makes controlling the deployment speed very difficult, whereas micromotors would have little difficulty with this task. However the position of the holding mechanism would need to be determined. It is envisioned that this problem could be overcome for micromotors by using a specifically designed encoder system or using the rise in current consumption as a system trigger.

## 4. Methods

There is a clinical need to target and treat pathologies of the GI tract such as ulcerative colitis, polyps, and Crohn's disease [39]. These pathologies are currently being treated by using conventional endoscopes in the upper and lower regions of the GI tract but the middle section, the jejunum, and ileum are only reachable through viewing a series of pictures from a WCE. Passive WCEs do not meet the clinical need to directly treat these pathologies of the small intestines. In order to examine or treat a specific location or feature within the GI tract a WCE would be required to stop. However it would still require small overall geometry to enable the capsule to pass through the junctions of the GI tract without becoming an obstruction.

### 4.1. Microrobot Concept

**Figure 3:**
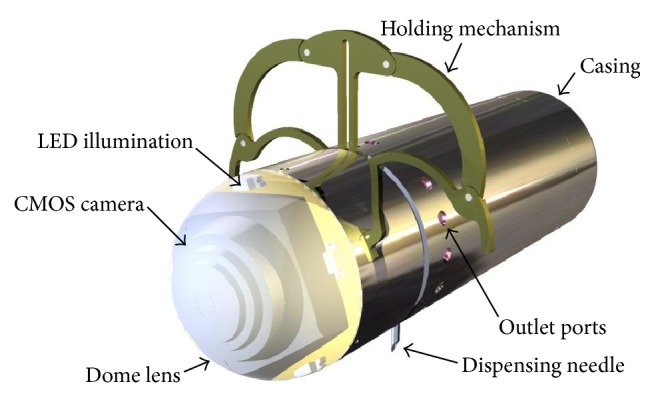
Concept microrobot capable of resisting peristaltic pressure through an integrated holding mechanism and delivering 1 mL of medication to a target site.

**Figure 4:**
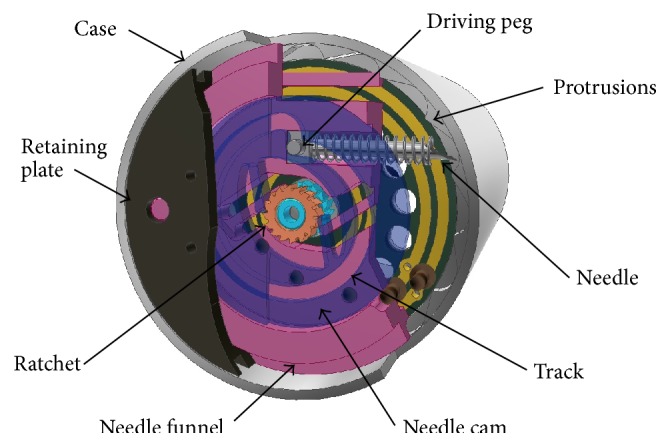
Needle positioning mechanism assembly with material removed for clarity. Needle shown in the fully retracted position.

Figure 3 represents a microrobot concept design capable of resisting peristaltic pressure through the deployment of an integrated holding mechanism and delivering a 1 mL dose of medication to a targeted site through the positioning of a needle. The needle has the ability to be positioned in a 360-degree envelope while simultaneously maintaining a diametrically opposite relationship with the holding mechanism; this novel feature guarantees needle penetration of the GI tract wall.  Figure 4 shows a section view through the component parts of the needle positioning mechanism.

Delivering a metered dose of medication to a site of interest is achieved through a novel mechanism utilising a cam, a needle funnel, two opposing ratchets, a needle, and a single micromotor manufactured by Namiki (Figure 4). The dimensions of the micromotor are *Ø*1.5 mm × 10.5 mm long. Orientating the micromotor along the central axis of the microrobot allows for simple coupling to the cam and needle funnel via two opposing ratchets. Driving the micromotor anticlockwise allows for the angular positioning of the needle, which has been fixed at one of 16 positions equally spaced. Driving the micromotor clockwise extends and retracts the needle. The novel positioning mechanism functions through the use of a series of ratchets engaging and disengaging to allow the various operations to be performed. A detailed evaluation of the targeting mechanism can be found in [40].

### 4.2. Microrobot Technical Specification

Conventional WCEs have a volume of 2.0 cm^3^; however the increased functionality requires a greater volume to house the mechanisms; therefore a volume of 3.0 cm^3^ has been chosen for the microrobot. Studies carried out by Connor et al. (2009) [41] have shown that capsules of dimensions *Ø*11.0 mm × 32.0 mm long are capable of being swallowed by subjects aged 18 to 65 years. Therefore the increased volume will have little impact on the majority of patients being able to swallow the microrobot or on its ability to navigate the junctions of the small intestine such as the ileocolic valve. The detailed specifications for the microrobot's performance are outlined in Table 4.

In order to realise a WCE with added functionality such as the ability to resist natural peristalsis or targeted drug delivery it is important to consider how these mechanisms can be operated.

**Figure 5:**
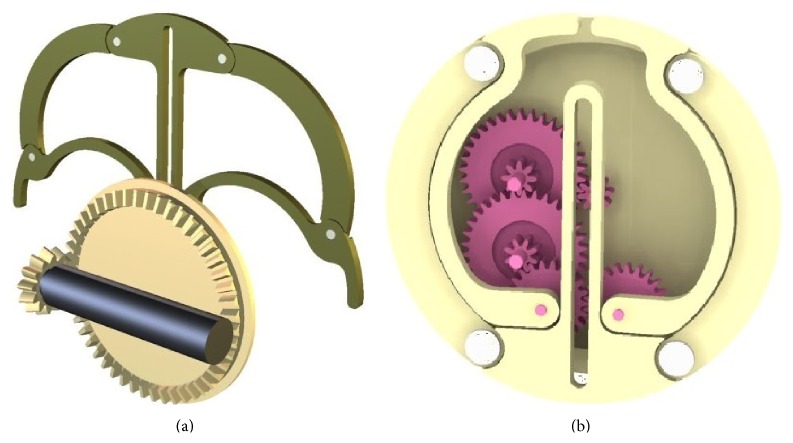
Fully extended holding mechanism and micromotor driving a bevel gear set (a); holding mechanism shown in the fully retracted position (b).

A single micromotor manufactured by Faulhaber (02/1) (Table 2) has been selected to drive the holding mechanism (Figure 5(a)). The micromotor's small package size *Ø*1.9 mm × 10.82 mm long allows the motor to be orientated perpendicular to the microrobot. The micromotor's novel configuration coupled with the bevel gear set allows rotation to be translated through 90 degrees and also a reduction in the micromotor's RPM while maximising the use of space (Figure 5(b)). The reduction in RPM will result in a multiplication of the micromotor's torque; this will give the legs the strength required to distend the GI tract wall and hold the microrobot in place [42].

Distending the GI tract wall in order to resist peristaltic contractions will result in forces being applied to the surface of the wall through the interaction of the legs with the wall. This interaction results in the possibility of the GI tract suffering from postoperative ileus. As a result of the GI tract being contacted the wall can cease to function. However postoperative ileus is usually associated with metabolic abnormalities or with the GI tract being handled such as in laparotomies or abdominal surgery and is not associated with endoscopy or with the use of WCE. The thin leg sections may also pose a potential risk of damaging the GI tract wall if external forces become too high. However a larger surface area could easily be integrated into the distal profile of the holding mechanism's legs to eliminate any potential trauma from operating the mechanism.

**Figure 6:**
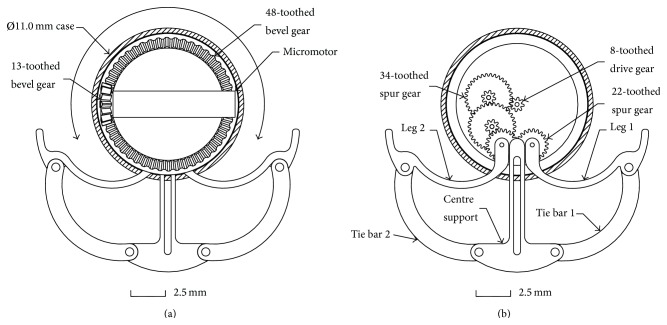
Fully extended holding mechanism: (a) 0.2 module 13-tooth and 48-tooth bevel gear set, (b) the 48-tooth bevel gear drives the central 0.1 module 8-toothed spur gear with the gear train running in a recess set in the bevel gear.

The bevel gear set comprises a 13-toothed drive gear, connected to the micromotor, and a 48-toothed follower gear (Figure 6(a)). The stability of the follower gear is derived from the casing of the microrobot and a cover which holds the micromotor and bevel gear in position. The 48-toothed bevel gear drives an 8-toothed spur gear (Figure 6(b)). The 8-toothed spur gear drives a gear train which runs inside a recess in the 48-toothed bevel gear. The last pair of 22-toothed gears drive the holding mechanism in and out via a connection between the gears and two legs.

### 4.3. Gear Train Parameters

The gear train reduces the 1,538 RPM output from the micromotor to 8.4 RPM; this allows the holding mechanism to be fully deployed in approximately 1.8 s. Based on an average transit time through the small intestines of 23 mm/min [19] the capsule would have travelled approximately 0.7 mm before the holding mechanism is fully deployed. The response time of the holding mechanism can be adjusted by reducing or increasing the number of teeth on the driver or follower gears.

The overall geometry of the microrobot and the gear module, which is the ratio of the pitch diameter to the number of teeth on the gear, has a direct influence on the dimensions of the gears. For example, the 48-toothed bevel gear has a module of 0.2; this results in an overall diameter of 10.0 mm which represents the maximum diameter that could fit within the microrobot. A module of 0.1 has been selected for the gear train as this facilitates maximum speed reduction yet minimises the use of space. The design parameters for the complete gear train are specified in Table 5.

## 5. Results

**Figure 7:**
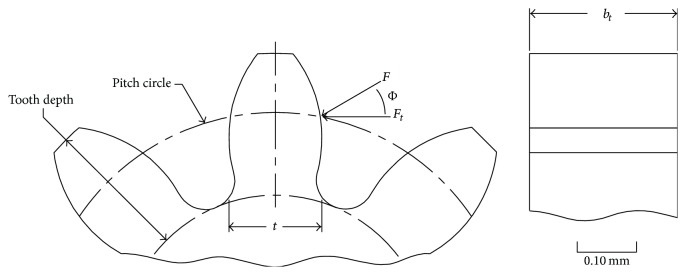
Gear tooth loading modelled as a cantilever beam.

The 0.1 module chosen for the gear train results in a micrometre gear tooth profile (Figure 7). It is therefore important to determine if the teeth can withstand the bending loads which they will be subjected to when the micromotor is operated at its maximum RPM. The Faulhaber (02/1) micromotor has been selected for the purpose of the gear train analysis. It has a two-stage 13 : 1 reduction gearbox which results in an output of 1,543 RPM and a torque of 0.15 mNm. The following sections analyse the loads which the teeth would be subjected to when the mechanism performs a full cycle.

### 5.1. Gear Tooth Loading

For the purposes of analysis the tooth can be modelled as a cantilever beam with an involute gear tooth profile. Figure 7 shows the forces acting at the pitch circle of the involute tooth profile and some additional relationships between features.

The contact angle between mating teeth is known as the pressure angle (Φ) and in this application it is set at an industry standard of 20 degrees for a spur gear.

### 5.2. Bending Stress Calculations

The stress figures determined by analysis are an important resource as they can be used to determine the material the gears are to be manufactured from and the manufacturing process.

The bending stress for a spur gear tooth or a straight toothed bevel gear can be obtained by using the Lewis formula which has been modified to take into consideration the contact impact of the gears through the addition of a velocity factor:

(3) $$\begin{array}{c} \sigma =\frac{F_{t}}{K_{v}b_{t}m_{n}y}, \end{array}$$

where *F*_*t*_ is the load applied to the tooth, *K*_*v*_ is the velocity factor, *b*_*t*_ is the face width, *m*_*n*_ is the normal module, and *y* is the Lewis form factor.

The assumptions made with the modified Lewis formula are that the full load is applied to a single tooth and the radial component force (*F*) is ignored and also that the force is distributed evenly over the full face width of the tooth and that the stress concentration effect of the tooth fillet is also ignored.

To calculate the load (*F*_*t*_) acting at the circular pitch

(4) $$\begin{array}{c} F_{t}=\frac{2000T_{f1}}{d_{1}}, \end{array}$$

where *T*_*f*1_ is the torque on the drive gear and *d*_1_ is the reference diameter of pinion and can be calculated by the number of teeth on the pinion (*z*) multiplied by the normal module (*m*_*n*_).

The velocity factor *K*_*v*_ compensates for the dynamic effect of the gears pitch line velocity and the manufacturing method used to produce the teeth profile. For a hobbed or shaped gear Barth's formula can be used to calculate the velocity factor:

(5) $$\begin{array}{c} K_{v}=\frac{3.54}{3.54+\sqrt{V}}, \end{array}$$

where *V* is the pitch line velocity and is calculated by

(6) $$\begin{array}{c} V=\frac{\pi dn}{60}, \end{array}$$

where *d* is the pitch diameter and *n* is the rotating speed of the gear in revolutions per minute.

The Lewis form factor (*y*) is a function of tooth shape and is independent of tooth size; it also does not take into consideration the stress raiser effect of the tooth fillet. It can be calculated as follows:

(7) $$\begin{array}{c} y=0.484-\frac{4.24}{z+6}, \end{array}$$

where *z* is the number of teeth on the gear.

The normal module (*m*_*n*_) of a gear is the ratio of the pitch diameter to the number of teeth on the gear. The module has a direct relation to the geometry of the teeth and the overall diameter (OD); it can be calculated as follows:

(8) $$\begin{array}{c} \text{OD}=\left(\;z+2\;\right)\times m_{n}. \end{array}$$

An example of the module's impact on the outside diameter can be shown using a module of 0.5 and 22 teeth as this would result in an outside gear diameter of 12.0 mm. If the same number of teeth was to be used with a 0.1 module the outside diameter would become 2.4 mm. Therefore the smaller the module the smaller the gear geometry which can be generated.

### 5.3. Tooth Bending Stress

Applying the modified Lewis formula (3) to the bevel gear set, which has a module of 0.2, results in a tooth bending stress of 3.57 Nmm^−2^ for the 13-toothed gear and 2.30 Nmm^−2^ for the 48-toothed gear. The calculated low figures for stress can be used to guide the design of the gears as the results suggest the gear set could be manufactured from a polymer such as PEEK. The benefit of making the 48-toothed gear from a polymer would be a simplified assembly as friction bushes can be eliminated by designing the gear to have bearing surfaces and reduced weight. However applying the formula to the next gear in the train results in significantly higher levels of bending stress.

**Figure 8:**
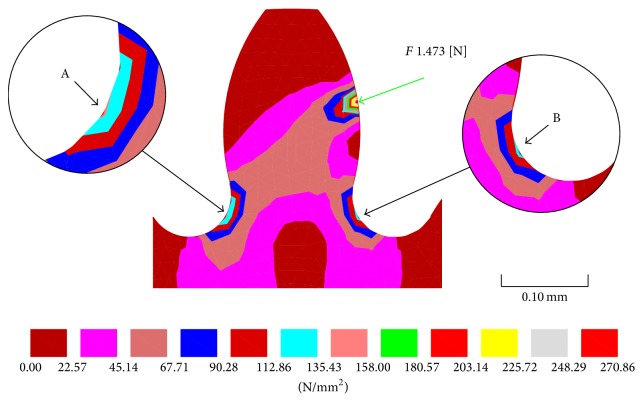
Von Mises FEA 2D isoareas analysis of a 0.1 module gear tooth profile with a radial component load of 1.473 N.

Applying the Lewis formula to the 8-toothed drive gear, which has a module of 0.1 and is connected to the 48-toothed bevel gear, yields a stress of 176.21 Nmm^−2^. At this level of stress it would result in a polymer tooth yielding; therefore a metallic gear would be required. The Lewis formula does not take into account the stress raising effect of the fillets or the stress distribution when the radial component load (*F*) is applied; therefore an FEA analysis has been performed to determine a more accurate level of stress distribution through the tooth (Figure 8).

Figure 8 shows a Von Mises FEA 2D isoareas analysis of a 0.1 module gear tooth profile with an applied radial component load of 1.473 N. The radial load *F* has been calculated from the applied load *F*_*t*_ and the pressure angle Φ. There is a distinct difference in the result for loading: Figure 8(A) shows the compressive stress to be 139.42 Nmm^−2^ while Figure 8(B) shows the tensile stress to be 121.69 Nmm^−2^. Although the FEA figures are lower than the calculated figure (176.21 Nmm^−2^) the FEA figures confirm that the 8 toothed gear must be manufactured from a metal rather than a polymer to ensure the teeth do not yield under load.

The Von Mises FEA analysis assumes a worst case scenario; that is, at any one time only one pair of teeth are in contact with each other and they take the total load. Generally two pairs of teeth are in contact; however this may drop to 1.5 depending on the degree of tooth truncation and inaccuracies in tooth profile due to the manufacturing process. Increasing the value of the face width or increasing the module would reduce the stress in the tooth; however increasing the module would result in an increase in the overall diameter of each gear in the set and hence the overall size of the gear train would increase. Also increasing the tooth thickness will influence the overall length of the microrobot due to the stack-up of dimensions. However increasing the size of the components would make manufacturing generally easier.

### 5.4. Gear Manufacture

**Figure 9:**
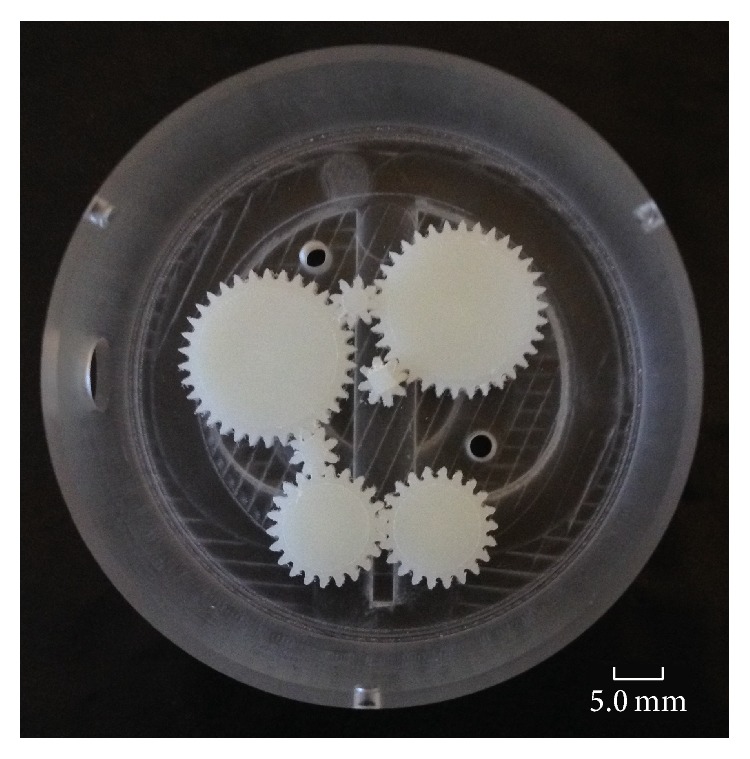
5 : 1 scale prototype of the holding mechanism's gear train.

There are a number of process routes which can be used to produce the holding mechanism's gear train; for example, hobbing, rapid prototyping, and wire EDM are all methods which could be employed to produce the spur gear set which drives the legs in and out. However, feature geometry, material selection, and manufacturing cost dictated that CNC milling was chosen.  Figure 9 shows a 5 : 1 scaled model of the complete gear train assembled in the gear box housing.

**Figure 10:**
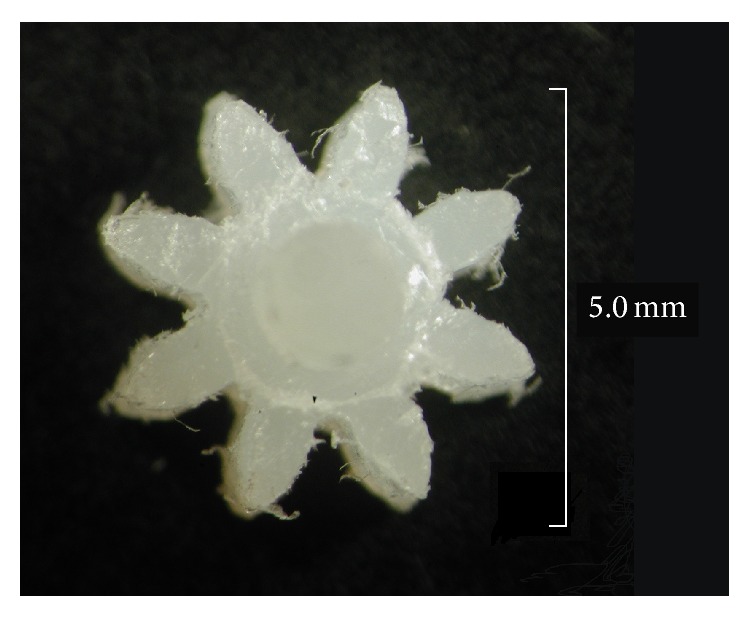
CNC milled 8-tooth spur gear manufactured from Nylon 6. The profile of the teeth is obscured by significant burring.

Prototyping the gears using a CNC milling machine allowed the use of small diameter end mills (*Ø*0.5 mm) to generate the tooth profiles and to achieve the tight root radii of the teeth. However the use of Nylon 6, which was selected for its mechanical properties, resulted in the gears having significant burrs owing to the manufacturing process. The burs can clearly be seen on the 8-tooth spur gear (Figure 10).

There are a number of manufacturing issues which can leave the gears with excessive burrs; for example, the end mill feed rate may be too fast or the wrong style of cutter may be selected. Cutter selection is very important as different materials have different requirements. Cutting plastic requires a sharp edged tool; therefore a hollow ground edged tool is preferable over a radially ground tool as it will be sharper and result in a cleaner edge.

### 5.5. Gear Measurements

Removing the burrs from all the gears in the gear train with a very sharp blade, such as a razor blade, allowed for inspection of the gears to confirm dimensional accuracy. Inspection of the 8-tooth spur gears was performed on a profile projector type PJ-300 manufactured by Mitutoyo at 10x magnification and a Mitutoyo 0–25 mm digital micrometer. The measured results have been collated in Table 6.

Comparing the measured dimensions with the design values shows that the average gear tooth thickness is greater than the nominal value and that the average tooth depth is shallower than nominal. The measured dimensions are well within the expected manufacturing tolerance limit for the given part; however the tendency for the teeth to be slightly bigger than required may pose problems with the gears meshing and running smoothly in the assembly.

### 5.6. Gear Train Performance

As can be seen from Figure 10 there are significant burrs on the top and bottom of the teeth profiles; these burrs are also evident on the larger gears in the train although not to such an extent. The burrs can influence the performance of the gear train in a number of ways; they can directly change the gear profile by being trapped between the rotating teeth and, in addition, the excess material will reduce the clearance between the gears; these two faults may result in a greater bending load being imposed on the tooth. The reduced clearance will also increase the friction within the system which places a greater load on the micromotor driving the train. Therefore it is important to remove the burrs from all the gears before operating the gear train. To overcome the issue of significant burring an alternative material such as stainless steel 316 could be used as a direct replacement for the gears.

The average thickness of the teeth (0.798 mm) suggests that the gear train would not run smoothly due to the reduced clearance; however on the larger gears the average thickness of the teeth was undersized (0.71 mm). This compensated for the thicker teeth and, once the burrs had been removed, resulted in a smooth running gear train. The slightly larger sized teeth on the smaller gears are an advantage in this instance as they can withstand a greater bending stress during operation.

### 5.7. Prototype Holding Mechanism

**Figure 11:**
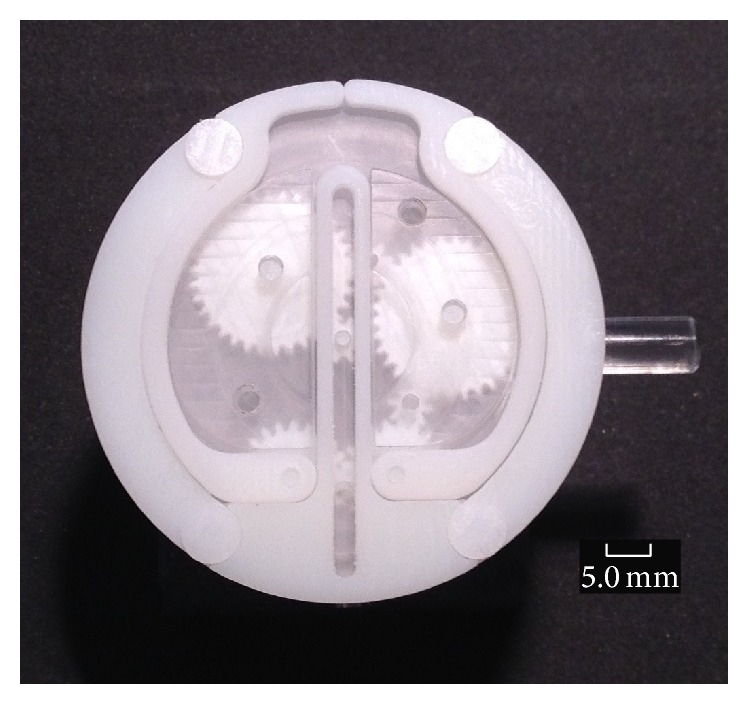
5 : 1 scale holding mechanism fully collapsed.

**Figure 12:**
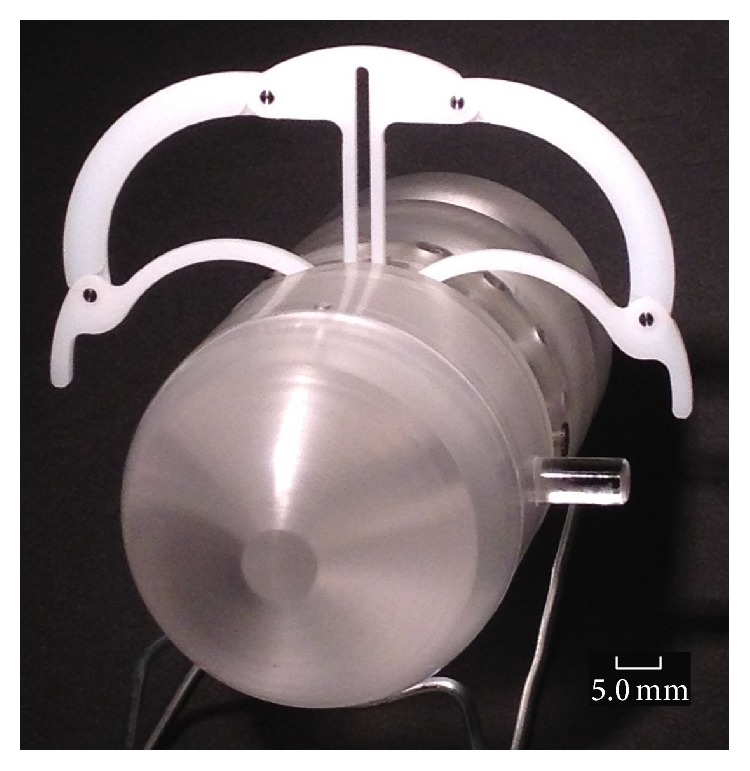
5 : 1 scale holding mechanism fully expanded.

The prototype holding mechanism comprises the micromotor, gear train (bevel gears and spur gears), and two legs connected to the gear train which drive the tie bars and centre support in and out (Figure 6). The legs, tie bars, and centre support are pinned together to allow them to pivot freely.  Figure 11 shows a section view of the fully assembled holding mechanism with some components removed for clarity and Figure 12 shows the complete microrobot with the holding mechanism fully expanded.

Figure 12 shows a prototype of the holding mechanism which can be manually operated through the rotation of a driveshaft connected directly to the gear train and which protrudes through the side of the gearbox; it also has a simplified spur gear train. The purpose of the simplified spur gear train was to enable manual functionality testing of the gears and the holding mechanism's legs and as such the number of turns required to operate the holding mechanism was kept to a minimum. However the gear train uses all the gears specified in the concept design (Figure 6 and Table 5).

## 6. Discussion

Delivering medication to a target site of interest in the GI tract requires an ability to overcome peristalsis. A prototype microrobot with an integrated holding mechanism has been presented which achieves this goal through the deployment of an expandable set of legs that are driven by a micromotor and a novel gearbox configuration. The holding mechanism has been designed for ease of manufacture and also for ease of assembly. However there were a number of issues with individual component parts and with the assembly of the parts which required addressing before a working prototype could be realised. For example, the material and manufacturing process chosen to prototype the gears resulted in gears with significant burrs which required removal before the gear train could be operated. Changing the gear material to a stainless steel would overcome this issue and also open up alternative manufacturing methods such as hobbing or wire EDM.

Manually operating the gearbox resulted in a smooth, free-running mechanism that took three and a half turns to fully expand the holding mechanism and three and a half turns to collapse it. However the calculated number of turns required to operate the mechanism is 2.6 turns. The difference in these two figures can be attributed to the backlash in the gear train which predominantly comprises the clearance between the gears, required for free running, and also the clearance between the gears' driveshaft and the housing. Reducing the backlash in future prototypes can be achieved through closer control of the manufacturing method chosen to produce the parts; for example, CNC milling and CNC turning could be used to produce the gears and gearbox. By applying tighter control over the dimensioning of the features the backlash in the gear train could be reduced. However the reduction in backlash would result in an increase in the manufacturing cost of the parts.

Producing prototypes which meet intended requirements will generally involve a compromise between part accuracy, surface finish, size, and cost. The selected manufacturing method will satisfy the majority of requirements; however the designer will ultimately decide which parameters are the most important and therefore which can be compromised.

## 7. Conclusion

In this paper we have presented the first holding mechanism of its kind for the purpose of resisting natural peristalsis in the GI tract. Exploitation of microactuators and conventional manufacturing techniques resulted in a holding mechanism which has been integrated into a WCE, occupying just 9% of the total available volume. The outcome of prototyping spur gears manufactured from Nylon 6 has highlighted the limitations with conventional CNC milling and material selection. This shows how adapting manufacturing methods to meet intended requirements can allow for radical changes in the capabilities of WCE systems in the future.

## Figures and Tables

**Table 1:** Comparison of WCE systems with increased functionality for the purpose of delivering therapy to the GI tract.

WCE	References	Status	Volume	Power	Motion	Vision	Therapy	Actuator
Park (2007)	[13]	Prototype	3.7 cm^3^	Te	A	—	—	Motor
Glass	[14]	Prototype	2.2 cm^3^	B	N, Sp	Camera	—	SMA wire/motor
Menciassi	[15]	Prototype	2.9 cm^3^	Te	N, Sp	Camera	—	SMA wire
Valdastri	[16]	Prototype	3.0 cm^3^	Te/B	A	Camera	—	Motor
Tognarelli	[17]	Prototype	2.9 cm^3^	B	N, Sp	CMOS	—	SMA flat/motor
Enterion	[18]	Commercial	2.9 cm^3^	M	N	—	L, P, S	Spring
InteliSite	[20]	Commercial	2.6 cm^3^	M	N	—	L	SMA wire
IntelliCap	[21]	Commercial	2.1 cm^3^	B	N	—	L	Microfluidic
Lambert	[22]	Prototype	3.5 cm^3^	B	N	—	L	Spring
Karargyris	[23]	Prototype	3.0 cm^3^	B	N	—	—	Spring
Bourbakis	[24]	Concept	3.0 cm^3^	B	N	Camera	—	Spring
Park (2008)	[26]	Prototype	1.9 cm^3^	B	N	Camera	Biopsy	Spring
Kong	[27]	Prototype	1.6 cm^3^	B	N	—	Biopsy	Spring
Peirs	[28]	Prototype	3.2 cm^3^	Te	N	—	Biopsy	Hydraulic
Gorini	[29]	Prototype	2.0 cm^3^	B	A	—	—	SMA wire
Kim	[30]	Prototype	3.8 cm^3^	B	A	Camera	—	SMA spring

—: data unavailable, A: active locomotion, B: battery, L: liquid medication, M: magnetism, N: natural peristalsis, P: powder medication, S: solids medication, Sp: static position, and Te: tethered.

**Table 2:** Power to volume merit index.

	Maxon	Namiki	Faulhaber	Squiggle
Power mW	30	60	130	340
Volume cm^3^	0.071	0.019	0.029	0.053

Merit index	0.422	3.157	4.482	6.415

**Table 3:** Actuator characteristics evaluation matrix.

Characteristics	Micromotor	Piezoelectric	SMA
Voltage	<5 V	>100 V	>2 V
Displacement	Unlimited	0.2%	>30%
Force	High	High	Medium
Speed	<1 kHz	~10 kHz	~0.1 Hz
Compactness	Poor	Good	Good
Motion type	Rotation	Linear	Linear

**Table 4:** Microrobot performance specification.

System feature	Requirement	Specification
Geometry	Microrobot volume	Maximum 3.0 cm^3^
	Maximum diameter	11.0 mm
	Maximum length	32.0 mm
	Maximum weight	5.0 g

Holding	Attaining equilibrium	Expansion
	Expand to a circumference	>53.0 mm
	Resist peristaltic contractions of	>26.9 g/cm circumferential
		>17.2 g/cm linear
	Actuator	Micromotor
	Deployment time	Maximum 2.5 s
	Holding time	Minimum 5 s

Targeting	Tracking:	RF
		Timed transit
	Telemetry	Bidirectional
	Actuator	Micromotor
	Return to site of interest	±5.0 mm

Delivery	Delivering therapy	Liquid medication
	Delivery method	Needle
	Drug reservoir	1 mL
	Wall penetration	1.0 mm to 2.0 mm
	Needle penetration force	8.9 MPa
	Target location	±30 deg
	Actuator	SMA
	Response time	Maximum 5 s

**Table 5:** Bevel gear and spur gear design parameters.

	Number of teeth	Normal module	Outside diameter	Tooth depth	Tooth thickness	Face width
	(*z*)	(*m*_*n*_)	(OD)	(*h*)	(*t*)	(*b*_*t*_)
Bevel gear 1	13	0.2	3.0	0.48	0.314	0.7
Bevel gear 2	48	0.2	10.0	0.48	0.314	0.7
Drive gear	8	0.1	1.0	0.24	0.157	0.45
Follower gear	34	0.1	3.6	0.24	0.157	0.45
Leg drive gear	22	0.1	2.4	0.24	0.157	0.45

**Table 6:** Statistical measured data for the 8-tooth spur gears.

	Value	Average	Min.	Max.	SD
Outside diameter (OD)	5.0	5.032	5.02	5.04	0.008
Face width (*b*_*t*_)	2.25	2.291	2.28	2.30	0.009
Tooth thickness (*t*)	0.785	0.798	0.79	0.81	0.006
Tooth depth (*h*)	1.2	1.16	1.14	1.18	0.011
